# Acute kidney injury in critical care: complications of hemophagocytic lymphohistiocytosis

**DOI:** 10.3389/fimmu.2024.1396124

**Published:** 2024-06-18

**Authors:** Mengya Zhao, Yiming Guan, Jin Lin, Yu Qiu, Shen Zhao, Meili Duan

**Affiliations:** ^1^ Department of Critical Care Medicine, Beijing Friendship Hospital, Capital Medical University, Beijing, China; ^2^ Department of Nephrology, Beijing Friendship Hospital, Capital Medical University, Beijing, China

**Keywords:** hemophagocytic lymphohistiocytosis, acute kidney injury, sepsis, critical care, intensive care unit

## Abstract

Hemophagocytic lymphohistiocytosis (HLH) is an immune dysfunction characterized by an exaggerated and pathological inflammatory response, potentially leading to systemic inflammatory reactions and multiple-organ failure, including renal involvement. HLH can be classified as primary or secondary, with primary HLH associated with genetic mutations affecting cell degranulation capacity, and secondary HLH often linked to infections, tumors, and autoimmune diseases. The pathogenesis of HLH is not fully understood, but primary HLH is typically driven by genetic defects, whereas secondary HLH involves the activation of CD8+ T cells and macrophages, leading to the release of inflammatory cytokines and systemic inflammatory response syndrome (SIRS). The clinical presentation of HLH includes non-specific manifestations, making it challenging to differentiate from severe sepsis, particularly secondary HLH due to infections. Shared features include prolonged fever, hepatosplenomegaly, hematopenia, hepatic dysfunction, hypertriglyceridemia, and hypofibrinogenemia, along with histiocytosis and hemophagocytosis. However, distinctive markers like dual hemocytopenia, hypertriglyceridemia, hypofibrinogenemia, and elevated sCD25 levels may aid in differentiating HLH from sepsis. Indeed, no singular biomarker effectively distinguishes between hemophagocytic lymphohistiocytosis and infection. However, research on combined biomarkers provides insights into the differential diagnosis. Renal impairment is frequently encountered in both HLH and sepsis. It can result from a systemic inflammatory response triggered by an influx of inflammatory mediators, from direct damage caused by these factors, or as a consequence of the primary disease process. For instance, macrophage infiltration of the kidney can lead to structural damage affecting various renal components, precipitating disease. Presently, tubular necrosis remains the predominant form of renal involvement in HLH-associated acute kidney injury (HLH-AKI). However, histopathological changes may also encompass interstitial inflammation, glomerular abnormalities, microscopic lesions, and thrombotic microangiopathy. Treatment approaches for HLH and sepsis diverge significantly. HLH is primarily managed with repeated chemotherapy to eliminate immune-activating stimuli and suppress hypercellularity. The treatment approach for sepsis primarily focuses on anti-infective therapy and intensive symptomatic supportive care. Renal function significantly influences clinical decision-making, particularly regarding the selection of chemotherapy and antibiotic dosages, which can profoundly impact patient prognosis. Conversely, renal function recovery is a complex process influenced by factors such as disease severity, timely diagnosis, and the intensity of treatment. A crucial aspect in managing HLH-AKI is the timely diagnosis, which plays a pivotal role in reversing renal impairment and creating a therapeutic window for intervention, may have opportunity to improve patient prognosis. Understanding the clinical characteristics, underlying causes, biomarkers, immunopathogenesis, and treatment options for hemophagocytic lymphohistiocytosis associated with acute kidney injury (HLH-AKI) is crucial for improving patient prognosis.

## Introduction

Hemophagocytic syndromes (HPS), also known as hemophagocytic lymphohistiocytosis (HLH), represent an immune dysfunction characterized by an exaggerated and pathological inflammatory response, resulting in hypercytokinemia and potential multiorgan failure. This condition can lead to significant damage in target organs, with acute kidney injury (AKI) being particularly noteworthy as a strong predictor of poor prognosis. Morbidity in HLH patients, reaching up to 50%, underscores the severity of the disease (1–4). Given its rapid progression and high mortality rates (5, 6), early diagnosis and intervention are pivotal for improving patient outcomes.

Recognizing the critical role of renal involvement, especially AKI, it becomes imperative to comprehend the clinical presentation, diagnosis, and treatment of HLH. Identifying high-risk factors for AKI in HLH patients and promptly admitting them to intensive care units for intervention are essential components of patient care. Unfortunately, the early recognition of AKI in HLH patients is presently underestimated, with limited reported studies. A comprehensive understanding of the incidence, clinical features, and prognosis of AKI in HLH patients is still lacking. Addressing these gaps in knowledge is crucial for enhancing the overall management and prognosis of patients with HLH.

In summary, this article provides an overview of the clinical manifestations, underlying causes, biomarkers, immunopathogenesis, and management strategies for patients presenting with hemophagocytic syndrome complicated by acute kidney injury (AKI), intended to assist clinicians in their understanding and management of this condition.

## Clinical features

Hemophagocytic lymphohistiocytosis (HLH) is a rare and inadequately diagnosed disease with an unknown true prevalence due to the non-specific nature of its clinical presentation. Consequently, the epidemiology of HLH-associated acute kidney injury (HLH-AKI) remains poorly characterized.

First documented by Scott and Robb-Smith in 1939, HLH is a syndrome characterized by fever, hepatosplenomegaly, peripheral hypoplasia, and macrophage proliferation in the bone marrow and other hematopoietic organs (7). The initial familial case was reported by Farquhar in 1952 (8), and infection-associated HLH was recognized as a distinct clinical and pathologic feature in 1979 (9). The term “Hemophagocytic lymphohistiocytosis” was officially adopted by the International Histiocyte Association in 1991 (10).

The clinical presentation of HLH is heterogeneous, constituting a group of diseases marked by prolonged fever, hepatosplenomegaly, hematopenia, hepatic dysfunction, hypertriglyceridemia, and hypofibrinogenemia. It is characterized by histiocyte proliferation and increased hemophagocytosis. The HLH-2004 criteria (11) remain internationally recognized for diagnosis (**Table 1**) but are more tailored to pediatric cases and have limitations when applied to adults. The 2023 HiHASC guidelines (12) propose rapid identification markers, such as fever, decreased blood counts, and elevated ferritin levels; ferritin thresholds are not the same in adults and children. The guidelines recommend that the quick screen should be available within 6 h–12 h. Confirmation of HLH diagnosis and investigation into potential triggers or underlying causes should be completed within the subsequent 24 h–48 h. Among the scoring systems utilized to assess HLH probability in patients, the H-score is presently deemed most suitable for adults (13). It incorporates three clinical variables (underlying immunosuppression, fever, organomegaly), five routine blood tests (ferritin, triglycerides, aspartate aminotransferase, fibrinogen, and cytopenias), and one histopathologic factor (tissue phagocytosis), resulting in a total score of 337 points. The probability of diagnosing HLH exceeds 99% for scores exceeding 250, with a cutoff value of 169. With ongoing improvements in clinical diagnosis leading to increased case identification, secondary HLH is more frequently diagnosed. It often occurs in conjunction with various underlying disease states, particularly in patients with malignant tumors, severe infections, and various critical illnesses causing organ dysfunction, including renal impairment. HLH secondary to rheumatic diseases, is designated as macrophage activation syndrome (MAS). MAS, a subtype of HLH, represents a severe complication of rheumatic diseases, arising from the dysregulated activation and proliferation of T lymphocytes and macrophages. MAS, particularly with heightened phagocytic activity, presents a grave and potentially lethal condition. Rheumatic disorders implicated in MAS encompass systemic juvenile idiopathic arthritis (SJIA), systemic lupus erythematosus (SLE), Kawasaki disease (KD), and dermatomyositis, each characterized by clinical features indicative of HLH. Secondary HLH manifests through diverse etiologies, as outlined in **Table 2**, detailing the underlying diseases contributing to HLH development (14).

**Table 1:** Diagnostic criteria for HLH-2004.

Feature	Cutoff
Fever	
Splenomegaly	
Cytopenia	≥2 cell lines
Hemoglobin	<90 g/L (neonates <100 g/L)
Platelets	<100 × 10^9^/L
Neutrophils	<1 × 10^9^/L
Hypertriglyceridemia or hypofibrinogenemia	≥265 mg/dL (≥3 mmol/L) ≤150 mg/dL (≤1.5g/L)
Hemophagocytosis	Bone marrow, other tissues
Hyperferritinemia	≥500 ng/mL (≥500 µg/L)
Reduced or absent natural killer-cell cytotoxicity	
Elevated soluble CD25 (soluble IL-2 receptor)	≥2,400 U/mL

**Table 2:** HLH-related secondary diseases.

Type	Diseases
Rheumatologic/autoimmune	Systemic juvenile idiopathic arthritis (SJIA) Adult onset Still disease (AOSD) Systemic lupus erythematosus (SLE) Sjogren’s syndrome Inflammasomopathies (e.g., dermatomyositis, inflammatory bowel disease) Kawasaki disease
Malignant	Hematologic (e.g., leukemia, lymphoma) Non-Hematologic
Infections	Viral [Epstein–Barr virus (EBV), cytomegalovirus (CMV), Herpes virus, influenza virus, rotavirus, hemorrhagic fever virus, human immunodeficiency virus (HIV)] sepsis-associated HLH (SAHS) Bacterial (Rickettsia, mycobacteria) Parasitic (e.g., Leishmania) Fungal (e.g., histoplasmosis)
Others	Pregnancy Drugs Graft-versus-host disease

However, discerning patients with hemophagocytic lymphohistiocytosis (HLH) from those experiencing severe sepsis presents a formidable clinical challenge. While experienced hematologists typically conduct early screening for suspected HLH cases, routine assessments for the majority of critically ill patients with similar clinical presentations rarely include specialized tests such as sCD25 and NK cell activity. This limited utilization of advanced diagnostic tools may result in the oversight or delayed diagnosis of HLH in a subset of patients, contributing to the rapid progression of the syndrome and elevated rates of morbidity and mortality. Incorporating these specialized tests into routine evaluations for critically ill patients with risk factors could enhance diagnostic accuracy, facilitating more timely interventions and potentially improving outcomes in cases of HLH.

Secondary HLH, particularly when induced by infection, closely aligns with the definition of sepsis, manifesting as organ dysfunction triggered by a cytokine storm. Due to their overlapping features, approximately one-fifth of septic patients may exhibit characteristics resembling HLH (15). In cases of septic shock, misdiagnosis with HLH is not uncommon (16) and the diagnosis of HLH is not sufficient for patients admitted to the ICU (17). Routine diagnostic evaluations of patients with systemic inflammatory syndromes at the Mayo Clinic have revealed that the prevalence of HLH in sepsis patients can be as high as 1 in 2000 (18). Notably, the phenomenon of tissue phagocytosis lacks sensitivity and specificity, making it challenging to differentiate between secondary HLH and sepsis. Phagocytosis is also observed in a subset of sepsis cases, complicating the distinction between phagocytosis directly induced by sepsis versus that triggered by sepsis-associated secondary HLH (17, 19, 20). In a multinational study involving 362 patients with systemic juvenile idiopathic arthritis (JIA) complicated by macrophage activation syndrome (MAS), phagocytosis was identified in only 60.7% of patients upon bone marrow examination (21). This underscores the complexity of the relationship between infection and HLH. The intricate relationship between HLH and sepsis is compounded by HLH’s inherent immune dysregulation. Moreover, chemotherapy and immunosuppressive treatments exacerbate mucosal damage, intensify immune dysfunction, and induce cytopenias, rendering patients more vulnerable to secondary infections. A study noted that 56% of HLH patients exhibited concurrent infections, with 67% succumbing directly to infectious complications (22).

HLH can be further complicated by infections during treatment due to immunosuppression. Additionally, infections can give rise to reactive hemophagocytic syndrome (RHS) (23), which may contribute to multiple-organ dysfunction syndrome (MODS). Both HLH and RHS may occur simultaneously, potentially triggered by the same cytokines. However, there is currently insufficient evidence to support a direct causal relationship between the two.

Regrettably, there is no specific diagnostic test for HLH and diagnosis continues to rely on clinical presentation. Efforts to enhance diagnostic precision in critically ill patients, particularly those with overlapping symptoms of HLH and sepsis, remain a critical area for future research.

Therefore, we propose several features that may distinguish HLH from sepsis:

1. Hematologic and immunologic markers:
  - Double hemocytopenia, hypertriglyceridemia, hypofibrinogenemia, and elevated sCD25 levels are less common in sepsis and may indicate true HLH (24).
  - Methemoglobinemia is a distinctive feature of HLH, and nearly all cases exhibit markedly elevated serum ferritin levels. However, significant variations in ferritin levels exist among patients with HLH, sepsis, infectious shock, and other conditions. The highest ferritin levels, observed in patients with HLH especially in deceased patients (25, 26), serve as well-recognized markers of HLH activity and prognosis (27, 28). Nevertheless, defining a precise cutoff value for serum ferritin remains controversial (25, 29).
  - Hypofibrinogenemia, particularly reported in children, may prove useful in identifying patients with sepsis/HLH overlap (30).
2. Renal manifestations in HLH-AKI:
  - Patients with HLH-AKI typically present with a clear clinical picture, including oliguria, azotemia, and nephrotic syndrome. Approximately 24% exhibit proteinuria in the nephrotic range (31), which may or may not be accompanied by evidence of phagocytic activity.
  - The Kidney Disease Improving Global Outcomes (KDIGO) (32) criteria are widely recognized as the standard for assessing renal impairment in critically ill patients (**Table 3**).

**Table 3:** Diagnostic criteria for KDIGO staging of AKI.

Stage	Feature	Cutoff
1	1.5–1.9 times baseline or ≥0.3 mg/dl (≥26.5 mol/l) increase	<0.5 ml/kg/h for 6 h–12 h
2	2.0–2.9 times baseline	<0.5 ml/kg/h for ≥12 h
3	3 times baseline or ≥4.0 mg/dl (≥353.6 mol/l) increase or initiation of RRT or in patients <18 years a decrease in eGFR <35 ml/min/1.73 m2	<0.3 ml/kg/h for ≥24 h or anuria ≥12 h

KDIGO, Kidney Disease Improving Global Outcomes; AKI, acute kidney injury; RRT, renal replacement therapy; eGFR, estimate glomerular filtration rate.

These proposed features aim to facilitate the differentiation between HLH and sepsis, providing clinicians with valuable clinical indicators to enhance diagnostic accuracy, especially in critically ill patients.

## Causes

The etiology of HLH-AKI, characterized by acute kidney injury complicated by phagocytic syndrome, can be broadly categorized into direct injury by inflammatory mediators, hemodynamic instability, tumor-associated renal injury, and the application of nephrotoxic drugs. It is challenging to pinpoint the specific cause of AKI, as it may involve multiple factors simultaneously.

Familial cases of HLH reported in the literature highlight activated macrophage infiltration as a direct consequence of specific renal parenchymal involvement (33). These cases confirm that HLH induces direct inflammatory damage to the kidneys. Patients in these familial cases responded well to high-dose intravenous glucocorticoids and continuous blood purification, showing a reversible transition from renal injury.

Secondary HLH, frequently triggered by infectious diseases, constitutes 44.5% of the etiology (34), with viruses, particularly herpesviruses like EBV and CMV, being predominant pathogens. Severe infectious cases can precipitate septic shock, resulting in renal hypoperfusion. Concurrently, the amplified cascade of inflammatory responses, triggered by severe infections, prompts the release of diverse inflammatory factors directly affecting the kidneys. This cascade leads to renal dysfunction, suggesting that the primary cause of acute kidney injury (AKI) is attributable to both renal hypoperfusion and the direct impact of inflammatory mediators on kidney function. Aulagnon et al. reported that hypoperfusion was the second-highest contributor to AKI etiology in 95 cases of secondary HLH, with most patients experiencing renal dysfunction at KDIGO stages 2 or 3 (31).

In patients with hematologic malignancies, AKI accounts for 35% of the etiology (34), primarily associated with B-cell lymphoma. Tumor-induced monoclonal gammopathy, renal injury due to malignant cell infiltration, and paraneoplastic tumors are potential AKI causes (35). Prerenal and acute tubular necrosis are common, often stemming from the direct effects of tumors, tumor complications, and chemotherapy.

Examination-related drugs, antitumor drugs, and anti-infective drugs used in diagnosis and treatment are frequently nephrotoxic, contributing to AKI in HLH patients. Previous exposure to nephrotoxic drugs has been reported in 72.3% of AKI patients (2), involving agents such as contrast agents, vancomycin, aminoglycosides, and nonsteroidal anti-inflammatory drugs, as well as methotrexate, siderophores, cyclophosphamide, and oxaliplatin. Recommended therapy for HLH include chemotherapy such as etoposide, which is cleared by the kidney.

## Pathophysiology

**Figure 1:**
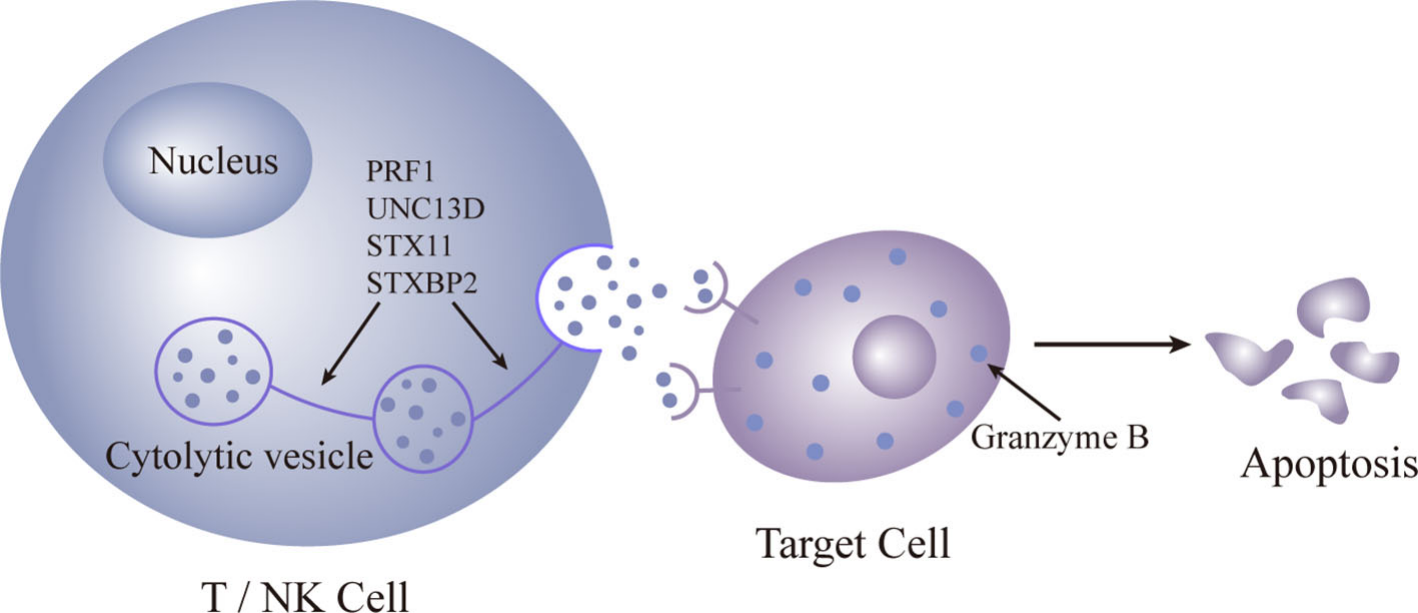
Pattern diagram of normal gene expression.

**Figure 2:**
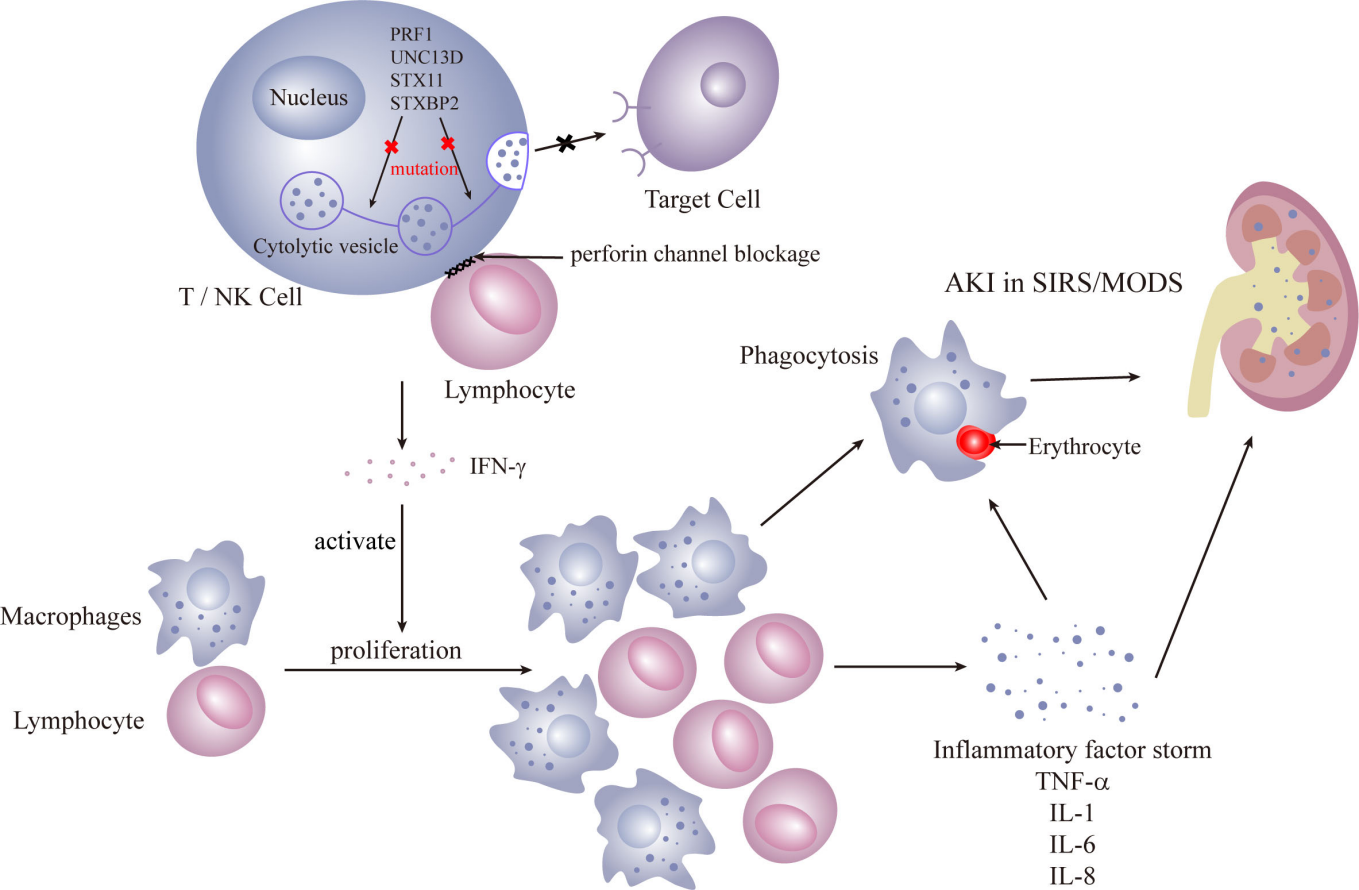
Primary HLH.

Primary HLH predominantly affects children, and immune dysfunction is typically irreversible. The primary cause is often autosomal recessive, resulting from defects in the release of cytoplasmic granules containing enzymes like perforin and granzyme B by cytotoxic T cells and NK cells. HLH leads to compromised cytotoxic T-cell and natural killer (NK) cell functionality. Pathogenic mutations in four genes (PRF1, UNC13D, STX11, and STXBP2) are identified (36–39), all impacting the perforin pathway crucial for lymphocyte cytotoxicity. These enzymes are crucial for cytotoxic activity, and their deficiency leads to uncontrolled proliferation of lymphocytes and macrophages. The subsequent phagocytosis is amplified by the excessive production of pro-inflammatory cytokines (**Figures 1**, **2**).

**Figure 3:**
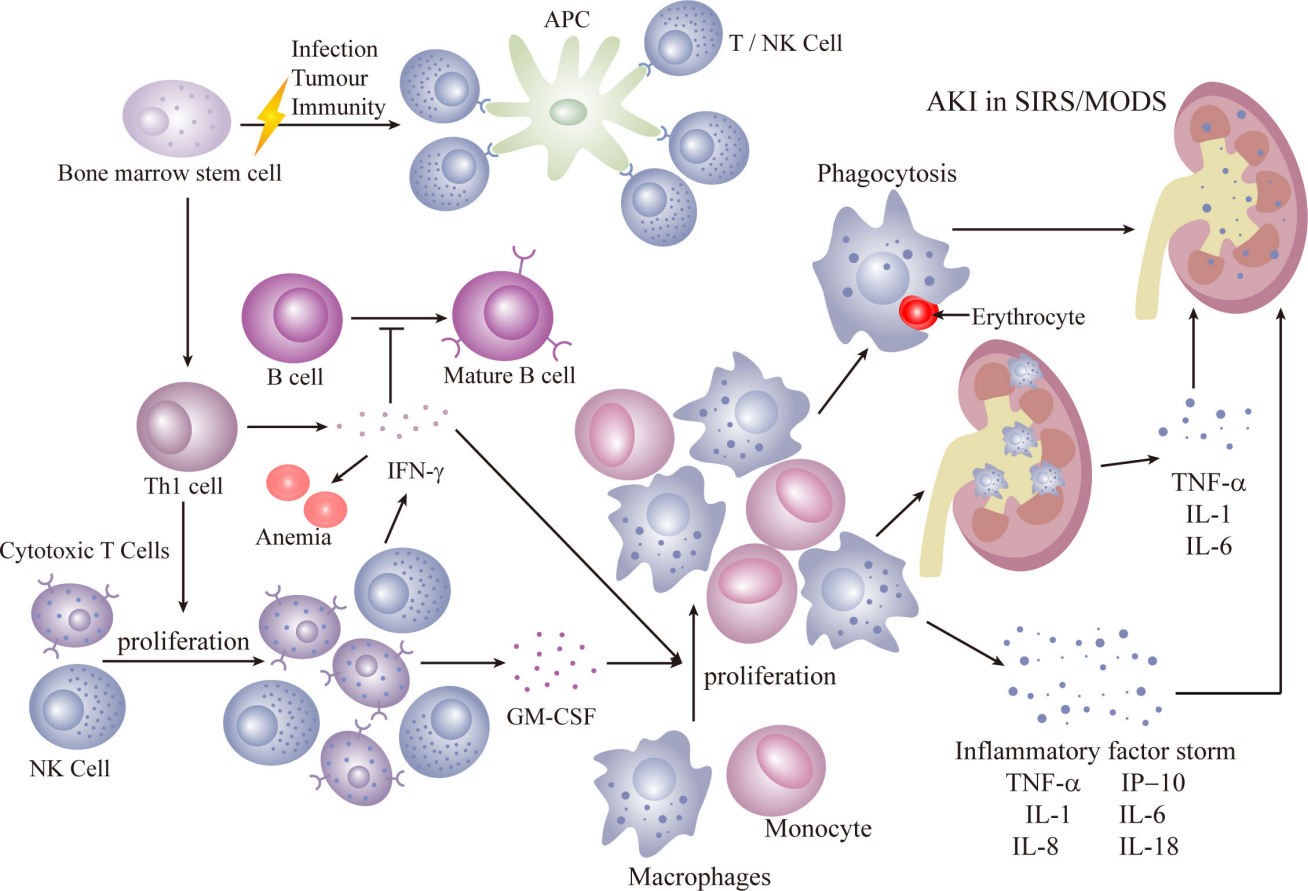
Secondary HLH.

Secondary HLH, more prevalent in adults, is triggered by infections, malignancies, and autoinflammatory/immune disorders. Infections are the most common cause, with the initial response coming from activation of antigen-presenting cells (APC), initiating a TH-1 response that induces cytotoxic T cells and NK cells to proliferate. Reversible dysfunction of NK or CD8+ T cells characterizes immune dysfunction. The release of interferon (IFN) and granulocyte-macrophage colony-stimulating factor (GM-CSF) by cytotoxic T cells leads to sustained expansion and activation of cytotoxic CD8+ T cells and macrophages, histiocyte proliferation, and infiltration into organs, including the kidneys. Renal infiltration of activated cytotoxic T cells and macrophages is recognized as a potential mechanism of AKI (40). Inflammatory factors such as TNF, IL-1, and IL-6 contribute to a cytokine storm, resulting in tissue damage, severe systemic inflammatory response syndrome (SIRS), and multiorgan failure (41) (**Figure 3**).

Both primary and secondary HLH involve an overactivation of the immune response, causing a cytokine storm that can lead to multiple-organ insufficiencies, including renal complications. Hallmarks of HLH include bone marrow infiltration and the presence of non-malignant macrophages in organs. Non-malignant macrophages, associated with phagocytic activity, can affect various kidney structures. In HLH-AKI, tubular involvement is prevalent (31), often manifesting as acute tubular necrosis, with an incidence of up to 62%. The majority of AKI cases progress to KDIGO 2 or KDIGO 3. Histopathological alterations reveal acute tubular necrosis accompanied by interstitial inflammation, observed in 45% of HLH patients (1). Tubulointerstitial damage may arise from hemodynamic shifts and coagulation disorders during the acute phase of severe sepsis. While glomerular involvement is infrequent, it can lead to collapsed glomerulopathy, focal segmental glomerulosclerosis, microscopic lesions, and thrombotic microangiopathy (3, 42–44). A direct deleterious effect of pro-inflammatory cytokines on the kidney is evident. Observations suggest that the direct toxic impact of pro-inflammatory cytokines (IL-6 and TNF-α) on renal tubular cells may be a mechanism in the development of AKI in HLH (1). Glomerular hemophagocytic macrophages may locally release nephrotoxic cytokines (45), inducing dysfunction in the local glomerular capillary wall and resulting in protein leakage, leading to proteinuria and renal dysfunction. Infiltration of the renal parenchyma by activated macrophages can contribute to reversible AKI (33).

## Biomarkers

Given the substantial morbidity and mortality associated with HLH-AKI and its non-specific clinical presentation, clinicians face a significant challenge in timely diagnosis and treatment. To enhance early identification of this patient group and improve clinical outcomes, various biomarkers have been reported. However, these biomarkers are often case-specific, lacking a standardized marker for accurate identification. Based on recent reports, we summarize the current understanding of these biomarkers as follows (**Table 4**).

1. Glycosylated ferritin:
  - HLH patients exhibit impaired cytotoxic function characterized by diminished perforin expression, leading to heightened cell lysis. Additionally, there is rapid ferritin synthesis, which may surpass the glycosylation process, or active suppression of glycosylation, resulting in reduced levels of glycosylated ferritin (46, 48–51).
  - Reduced percentage of glycosylated ferritin is considered a specific marker for confirming HLH diagnosis (46, 67).
  - Studies suggest that the pattern of released inflammatory cytokines may aid in differentiating HLH from infections and other causes of methemoglobinemia (48, 52, 60, 64, 68).
2. Ferritin (46, 47)
  - As a widely distributed globular protein, ferritin comprises 24 heavy chain (FtH) and light chain (FtL) subunits. Its expression escalates in both HLH and sepsis, with hyperferritinemia typically correlating with sepsis severity (69, 70). FtL may facilitate immunomodulation and mitigate septic acute kidney injury (AKI). Some studies indicate that ferritin levels ≥500 ng/ml aid HLH diagnosis (71), whereas others propose a threshold of >13,405 ng/ml for distinguishing infection from HLH (72). Zhang et al. put forth a diagnostic framework concerning ferritin (73).
  - Ferritin mediates a positive feedback loop involving the activation of inflammatory vesicles, resulting in increased production of IL-1 and IL-18. Elevated extracellular ferritin levels subsequently induce activation of innate immune cells while concurrently suppressing adaptive immune cell function (47).
  - Elevated plasma catalytic ferric ion levels were correlated with increased rates of death/requirement for renal replacement therapy, AKI, and in-hospital mortality. This association was observed in a study comprising 121 critically ill patients admitted to medical or surgical intensive care units (74).
3. IL-18 and new diagnostic parameters:
  - IL-18, synthesized primarily by activated macrophages and prominently expressed in cytotoxic lymphocytes, stimulates the secretion of additional pro-inflammatory cytokines. It augments the impact of IFN-γ production by lymphocytes, thereby amplifying the inflammatory response (47, 53, 60).
  - Ferritin, IL-18, and glycosylated ferritin are proposed as effective parameters for early HLH diagnosis.
  - A new scale combining IL-18 with the H-Score is considered more specific. IL-18 levels are reported to be higher in HLH caused by malignant diseases compared with infections (47). Chronic elevation of IL-18 has shown a significant correlation with HLH (60).
  - Furthermore, IL-18 has emerged as a potential novel biomarker for AKI. Studies indicate that IL-18-deficient mice exhibit protection against ischemia/reperfusion-induced AKI (75). Administration of IL-18-binding protein before ischemia/reperfusion injury has been shown to mitigate renal injury in rats (76).
4. Soluble interleukin 2 receptor (sCD25):
  - Reactive T-lymphocyte activation and proliferation, along with the expansion of transplanted lymphocytes, can lead to immune dysfunction and interference with normal immune processes (48, 52).
  - Serum sCD25 is another important marker reflecting immune activation in autoimmune diseases, tumors, and infections (55).
  - O Soluble CD25 (sCD25) can be directly shed from the surface of tumor cells. Higher levels of sCD25 are observed in patients with malignant tumors; excessively low levels (≤2,400 U/ml) of sIL-2r help exclude HLH (52).
5. CD38^high^/HLA-DR^+^ cell expression:
  - CD38high/HLA-DR+ cells are indicative of activated T cells. Increased CD38^high^/HLA-DR+ cell expression in T cells from HLH patients is proposed as a potential diagnostic marker. Expression in patients with sepsis is indistinguishable from that of healthy individuals (65).
  - Further evidence from large prospective studies is needed to validate its diagnostic utility.
6. Procalcitonin (PCT):
  - Procalcitonin (PCT), as a marker of inflammation, aids in distinguishing between bacterial and non-bacterial infections. It is frequently employed in the diagnosis of acute infections and for monitoring infection progression. PCT monitoring facilitates the evaluation of infection response and informs the development of anti-infective treatment strategies (73). The utilization of PCT for guiding antimicrobial therapy in severe infections and sepsis has been documented (77).
  - Serum procalcitonin levels exhibit notable elevation in individuals with severe bacterial infections compared with those with viral infections or non-specific inflammatory conditions (78). Research in patients with critical infections demonstrates that utilizing PCT to guide therapy effectively reduces antibiotic utilization and correlates significantly with improved mortality rates (79). Furthermore, studies involving patients with systemic lupus erythematosus (SLE) indicate that PCT can facilitate early detection of bacterial or fungal infections during the febrile phase of SLE (80), enabling differentiation between infectious etiologies and active SLE. In individuals with concurrent renal insufficiency, PCT serves as a reliable marker for bacterial infections (81, 82).
  - Several studies have noted a correlation between procalcitonin levels and ferritin, hinting at the possibility that PCT is involved in the cytokine pattern of HLH (83). Specific mechanisms underlying this relationship warrant further investigation in subsequent studies.

**Table 4:** HLH-related biomarkers.

Biomarkers	Results	Features	Pathogenesis
Ferritin (46, 47)	↑	• Early stage • High specificity • Secondary HLH	➢ Accelerated synthesis in the reticuloendothelial system ➢ Ferritin is released by splenic macrophages after phagocytosis by erythrocytes ➢ Protein leakage from damaged cells ➢ Ferritin mediates a positive feedback loop of inflammatory vesicle activation and IL-1, IL-18 production and high levels of extracellular ferritin, leading to innate immune cell activation and adaptive immune cell suppression
Glycosylated Ferritin (46, 48–51)	↓ ≤20%	• Early stage • High specificity	➢ Role of activated macrophages ➢ Cytotoxic functional impairment ➢ Reduced expression of perforin ➢ Massive cell lysis ➢ Ferritin synthesis is too fast to complete the glycosylation process/glycosylation process is actively downregulated
sCD25 (48, 52)	↑ ↑↑	• Early stage • Rheumatologic/autoimmune/infections • Higher levels in hematologic malignancies	➢ Activation of reactive T-lymphocytes ➢ Inhibits lymphocyte proliferation and interferes with immune function ➢ Released directly from the surface of tumor cells ◼ Products of activation of normal peripheral monocytes in response to tumor growth ◼ Products released by activated lymphoid cells infiltrating tumor tissue
IL-1β (53, 54)	↑	• sJIA highly relevant	➢ Produced by activated mononuclear macrophages ➢ Prototype products of inflammatory vesicle activation ➢ Activation of exogenous ATP via the P2X7 receptor causes activation of leukocytes and endothelial cells and production of inflammatory factors
IL-2 (55)	↑	• Malignant	➢ Produced by activated CD4 T cells ➢ Stimulates NK cells and CD8 T cells to lyse tumor targets
IL-6 (48, 56)	↑ ↑↑	• Early stage • A key role in cytokine storms	➢ Produced by macrophages and secreted with TNF-α and IL-1β during early inflammation ➢ Reduces perforin and granzyme B expression and inhibits cytotoxicity in NK cells ➢ Leads to increased inflammatory response to TLR ligands
IL-10 (48, 50, 57–59)	↑↑	• Early stage • Malignant/primary HLH/EBV-HLH	➢ Possesses potent anti-inflammatory properties, mainly signaling through the JAK-STAT pathway ➢ Interferes with the function of antigen-presenting cells and the expression of major histocompatibility complex-2 ➢ Inhibits T cell proliferation and cytokine secretion ➢ IL-10 levels determine immune regulation and the balance between inflammatory and humoral responses
IL-18 (47, 53, 60)	↑↑	• Early stage • High specificity • Malignant • More specific in combination with H-scores	➢ Produced by activated macrophages and most strongly expressed in cytotoxic lymphocytes ➢ Induces the release of other pro-inflammatory cytokines ➢ Amplifies IFN-γ production by lymphocytes
IFN-γ (2, 47, 48, 56, 61, 62)	↑↑	• Early stage • Malignant • Key drivers for HLH	➢ Type II interferon secreted by T lymphocytes and NK cells in Th1-mediated immune response ➢ Activates monocytes and macrophages and converts them to phagocytes ➢ Impairs B-cell development and maturation ➢ Induces secretion of inflammatory factors in HLH, high levels of IFN-γ lead to severe HLH
CXCL9 (47)	↑↑	• Malignant	➢ Downstream effector chemokine of IFN-γ, secreted by myeloid cells after exposure to IFN-γ ➢ Mediates lymphocyte activation
TNF-α (53, 56, 63)	↑	• Non-core cytokines	➢ Produced by macrophages and monocytes ➢ Induces further macrophage activation ➢ Increases adhesion of vascular endothelial cells to NK cells/direct toxic effect on NK cells, negatively regulates NK cell activity
M-CSF	↑	• Inflammatory/autoimmune diseases	➢ Promotes monocyte/macrophage proliferation ➢ Involved in a variety of inflammatory and autoimmune diseases
IP-10 (64)	↑↑	• Early stage • Distinguish between HLH and sepsis	➢ Produced by IFN-γ-activated macrophages ➢ Chemoattractant of activated T cells, especially IFN-γ producing Th1 cells, attracts Th1 cells to activated macrophages via CXCR3 ➢ Th1 cells produce IFN-γ, which further activates macrophages to produce IP-10
CD38^high^/HLA-DR+ cells (65)	↑↑	• Distinguish between HLH and sepsis • Significant in HLH	➢ Characteristics of activated T cells, CD81 T cells are most obvious ➢ Activated T cells are type I polarized, proliferate, and show recent and sustained activation
sCD163 (66)	↑↑	• HLH/MAS/sepsis/SAHS • Distinguish between HLH and sepsis • Significant in HLH	➢ Expression is restricted to cells of the monocyte-macrophage lineage, a marker of macrophage activation ➢ In response to inflammatory stimuli, macrophages are activated and the extracellular portion of the protein is shed into the plasma ➢ sCD163 levels correlate with neutrophil elastase levels due to phagocytosis and destruction by granulocytes

HLH, hemophagocytic lymphohistiocytosis; sCD25, soluble interleukin 2 receptor; sJIA, systemic juvenile idiopathic arthritis; NK cells, natural killer cells; TNF-α, tumor necrosis factor-α; EBV, Epstein–Barr virus; JAK-STAT, Janus kinase-signal transducer and activator of transcription; TLR, Toll-like receptors; IFN-γ, interferon-γ; M-CSF, granulocyte-macrophage colony-stimulating factor; MAS, macrophage activation syndrome; SAHS, sepsis-associated HLH. “↑” indicates elevated. “↑↑” indicates significantly elevated.

No singular biomarker effectively distinguishes between HLH and sepsis; however, some studies explore combined biomarkers providing insights into the differential diagnosis. A distinctive cytokine profile characterized by markedly elevated levels of IFN-γ and IL-10, coupled with moderately elevated IL-6 levels, exhibits high diagnostic accuracy for HLH (48). This cytokine pattern aids in distinguishing HLH from infection. Additionally, the cytokine profile consisting of IL-18, IL-1β, and IFN-γ demonstrates good sensitivity and specificity in identifying severe infections (84).

In conclusion, whereas various biomarkers show promise in aiding HLH diagnosis, the lack of a standardized marker necessitates ongoing research and validation through large-scale prospective studies.

## Treatment

**Figure 4:**
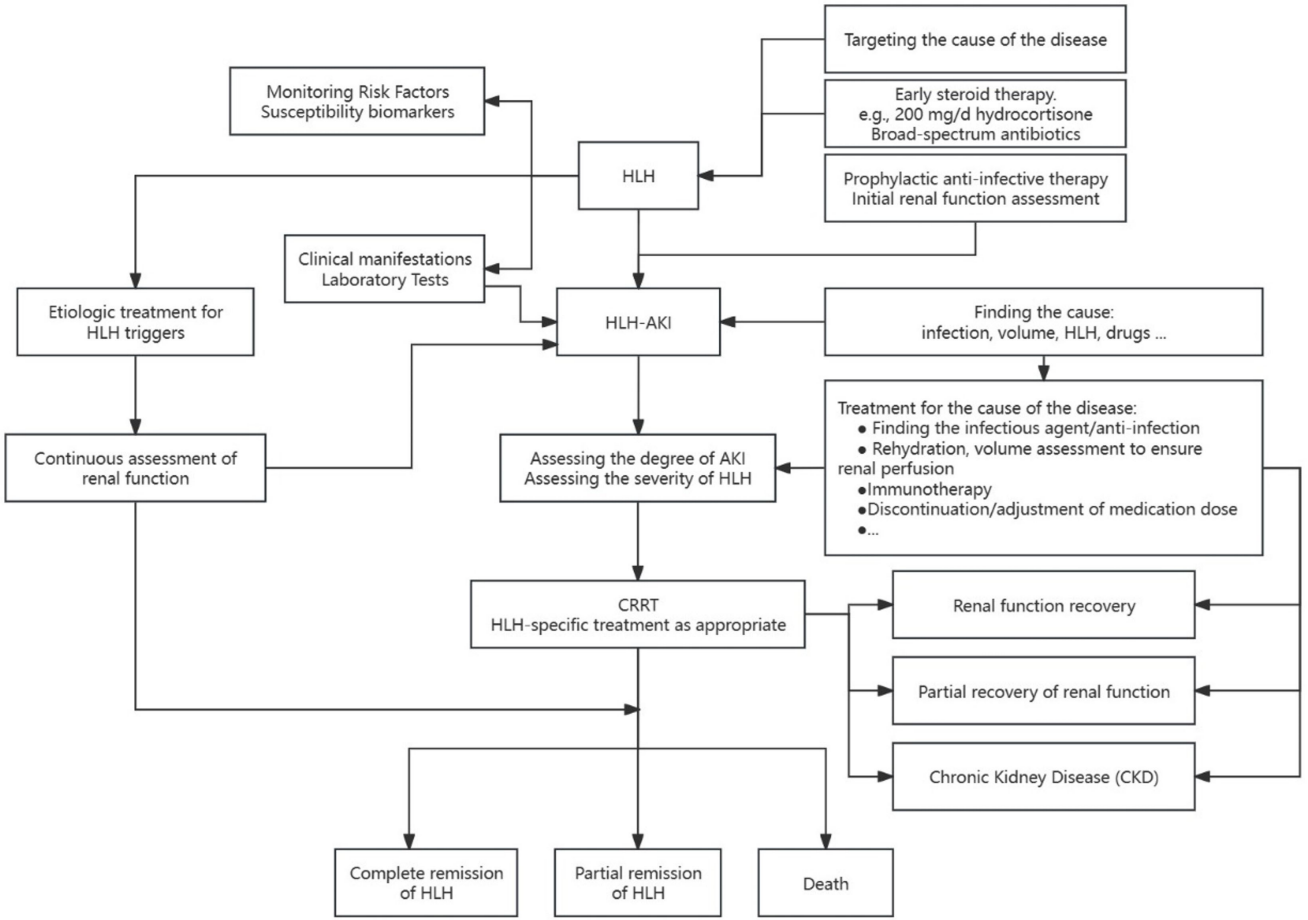
Management of patients with HLH-AK.

Despite the similarities between HLH and sepsis, including multiorgan dysfunction and similar clinical features, their treatment approaches differ significantly. HLH treatment focuses on repetitive chemotherapy to eliminate immune activation stimuli and suppress hypercytokinemia, patients with rapid clinical deterioration are the target population for HLH chemotherapy, and potential triggers need to be identified and treated in patients whose clinical status is stable, whereas sepsis treatment primarily relies on anti-infective therapy and intensive symptomatic care (**Table 5**, **Figure 4**).

**Table 5:** Treatments of HLH and sepsis.

Treatments	HLH	Sepsis
Treatment of the causes	Primary HLH HLH-1994/2004 (Dexamethasone, etoposide, cyclosporine) allo-HSCT	Antibiotics • Early • rapid
	Secondary HLH • Infections: treatment of the primary disease, with or without chemotherapy as appropriate • Rheumatic diseases: immunosuppressive therapy/chemotherapy • Tumors: treat primary tumor, control inflammatory response	
Immunosuppressive therapy	Strong	Weaker
Others	Rucotinib Anakinra IVIG/corticosteroids IL-6 blockers	Organ support • Mechanical ventilation • Kidney replacement therapy • vasopressor support • Extracorporeal membrane oxygenation (ECMO) • Blood transfusion therapy

allo-HSCT, allogeneic hematopoietic stem cell transplantation; IVIG, intravenous immunoglobulin.

### HLH treatment

- Cause-specific therapy involves induction therapy, following the HLH-1994/2004 regimen with dexamethasone, etoposide, with or without cyclosporine, and allogeneic hematopoietic stem cell transplantation (allo-HSCT) for familial, persistent, and recurrent HLH (11).
- Treatment protocols are largely based on pediatric studies, and their applicability to adults is uncertain. It has also been suggested that the intensity of immunosuppression in pediatric HLH94 protocols is neither appropriate nor necessary for most adults (21, 85). Weaker immunosuppressive and cytotoxic methods may be preferable for infection-related HLH (86).
- Ruxolitinib, a JAK1 and JAK2 inhibitor, and other treatments like interleukin-1 antagonist (anakinra), intravenous immunoglobulin (IVIG), and/or corticosteroids, have shown promise (87–93).
- No standardized protocols exist, requiring individualized treatment plans based on factors such as disease progression, inflammatory response, triggers, and concomitant diseases, with current experience relying primarily on observational studies and case reports.

### Supportive interventions in critically ill patients

- Critically ill HLH patients, especially those with acute kidney injury, may require aggressive supportive interventions (94) such as broad-spectrum antibiotics, vasopressors, renal replacement therapy, mechanical ventilation, blood product transfusions and coagulopathy management, and even extracorporeal membrane oxygenation (ECMO) (5, 95, 96). Patients who fail to respond to initial therapy typically experience a poorer prognosis, often accompanied by a higher 30-day mortality rate (97).
- Early steroid therapy is valuable in stabilizing the disease, particularly in primary HLH and previously untreated secondary HLH (94), and that steroid therapy should not be delayed, especially in the presence of organ dysfunction. In difficult-to-diagnose cases, short-term treatment with corticosteroids is suggested (71), along with broad-spectrum antibiotics, as 200 mg/day of hydrocortisone has been shown to be beneficial in patients with sepsis shock (98),whereas if HLH is suspected, it is even more recommended that higher doses of corticosteroids be administered as early as possible, before the patient goes into sepsis shock. In acutely critically ill patients, the administration of full-dose chemotherapy regimens for treating HLH is suboptimal and can even lead to fatalities in some instances (99, 100). Consequently, guidelines advocate for early intervention directed at mitigating the cytokine storm through the use of reduced doses of etoposide (101).
- Mortality rates among critically ill patients with HLH have shown no recent decrease, and several factors may contribute to this trend. These include high-intensity treatment leading to severe immunosuppression, which is secondary to severe infections. The inappropriate application of drugs, such as in cases of combined hepatic and renal dysfunction, emphasizes the need to reduce the dose of etoposide rather than discontinuing the drug (18, 94, 102–104). Additionally, delayed diagnosis and treatment could also play a role in the sustained mortality rates.

### Continuous renal replacement therapy

- Continuous renal replacement therapy (CRRT) serves as a crucial adjunctive support for renal function replacement in patients with HLH-AKI, contributing to internal environment stability and inflammatory factor removal.
- There are no standardized protocols for the treatment of HLH-AKI, either in case reports or in clinical studies, and the selection of the timing of treatment, the application of different filters, the formulation of the therapeutic dose, and the assessment of the end of treatment are all based on the experience of individual clinical hospitals, and the choices vary widely from patient to patient and from physician to physician (26, 66).
- While continuous renal replacement therapy (CRRT) has been reported to provide stabilization in patients, the persistently high morbidity and mortality rates may be associated with factors such as the severity of the disease, the timeliness of diagnosis, and the intensity of treatment. Moreover, the long-term prognosis of this patient cohort remains uncertain, with potential progression to chronic kidney disease (CKD) (40).

In conclusion, while advancements have been made in HLH treatment strategies, challenges persist, emphasizing the need for individualized approaches and ongoing research. The effectiveness of CRRT in HLH-AKI underscores its role as a supportive measure, but further studies are warranted to establish standardized protocols.

## Conclusions

Timely diagnosis plays a pivotal role in the identification and management of patients with hemophagocytic lymphohistiocytosis (HLH), facilitating the reversal of renal impairment and creating a therapeutic window for HLH intervention. Collaborative efforts involving hematologists, intensivists, and nephrologists are essential for a comprehensive understanding of HLH pathogenesis, early identification of critically ill patients, prompt initiation of immunomodulatory therapy, and the optimization of organ supportive care. These measures hold the potential to enhance the prognosis and increase the complete remission rate among patients with HLH. Future research endeavors should focus on further unraveling the complexities of HLH and refining multidisciplinary approaches for improved patient outcomes.

## Author contributions

MZ: Writing – original draft, Conceptualization. YG: Writing – original draft, Visualization. JL: Writing – review & editing, Supervision. YQ: Writing – review & editing, Validation. SZ: Writing – review & editing, Validation, Supervision. MD: Writing – review & editing.
